# Supplementary material for: Heparin-based hydrogel scaffolding alters the transcriptomic profile and increases the chemoresistance of MDA-MB-231 triple-negative breast cancer cells
Source: Biomater Sci. 2020 Feb 13;8(10):2786–96. doi: 10.1039/c9bm01481k (PMC7497406; doi:10.1039/c9bm01481k)
Supplement: Supplementary file 2 [file BM-008-C9BM01481K-s002.zip › Supplementary File 4/HP-BhydrogelvControl/Pathways/my_analysis.Gsea.1545201079907/HALLMARK_G2M_CHECKPOINT.html]

Details for gene set HALLMARK\_G2M\_CHECKPOINT[GSEA]

|  || Dataset | expr.class.cls#HYD\_versus\_CONTROL.class.cls#HYD\_versus\_CONTROL\_repos |
| Phenotype | class.cls#HYD\_versus\_CONTROL\_repos |
| Upregulated in class | CONTROL |
| GeneSet | HALLMARK\_G2M\_CHECKPOINT |
| Enrichment Score (ES) | -0.2563261 |
| Normalized Enrichment Score (NES) | -1.2305275 |
| Nominal p-value | 0.026737968 |
| FDR q-value | 0.12067926 |
| FWER p-Value | 0.697 |
Table: GSEA Results Summary

  

Fig 1: Enrichment plot: HALLMARK\_G2M\_CHECKPOINT      
 Profile of the Running ES Score & Positions of GeneSet Members on the Rank Ordered List

  

| PROBE | DESCRIPTION (from dataset) | GENE SYMBOL | GENE\_TITLE | RANK IN GENE LIST | RANK METRIC SCORE | RUNNING ES | CORE ENRICHMENT || 1 | NUMA1 | na |  |  | 566 | 2.257 | -0.0102 | No |
| 2 | ABL1 | na |  |  | 912 | 1.940 | -0.0115 | No |
| 3 | PAPD7 | na |  |  | 1263 | 1.707 | -0.0151 | No |
| 4 | RBM14 | na |  |  | 1766 | 1.462 | -0.0289 | No |
| 5 | ODF2 | na |  |  | 1984 | 1.369 | -0.0284 | No |
| 6 | TNPO2 | na |  |  | 1997 | 1.364 | -0.0172 | No |
| 7 | E2F4 | na |  |  | 2267 | 1.252 | -0.0205 | No |
| 8 | UPF1 | na |  |  | 2409 | 1.205 | -0.0175 | No |
| 9 | ILF3 | na |  |  | 2804 | 1.084 | -0.0288 | No |
| 10 | SFPQ | na |  |  | 2890 | 1.065 | -0.0240 | No |
| 11 | SMAD3 | na |  |  | 2896 | 1.064 | -0.0150 | No |
| 12 | CDC25B | na |  |  | 3153 | 0.999 | -0.0198 | No |
| 13 | RASAL2 | na |  |  | 3579 | 0.898 | -0.0344 | No |
| 14 | DDX39A | na |  |  | 3637 | 0.885 | -0.0297 | No |
| 15 | MYC | na |  |  | 3771 | 0.854 | -0.0293 | No |
| 16 | CHMP1A | na |  |  | 3854 | 0.842 | -0.0263 | No |
| 17 | POLE | na |  |  | 3906 | 0.832 | -0.0218 | No |
| 18 | CHAF1A | na |  |  | 3907 | 0.831 | -0.0146 | No |
| 19 | RAD54L | na |  |  | 3989 | 0.814 | -0.0118 | No |
| 20 | POLA2 | na |  |  | 4005 | 0.811 | -0.0055 | No |
| 21 | E2F2 | na |  |  | 4167 | 0.776 | -0.0073 | No |
| 22 | UCK2 | na |  |  | 4278 | 0.756 | -0.0065 | No |
| 23 | E2F1 | na |  |  | 4422 | 0.730 | -0.0077 | No |
| 24 | TLE3 | na |  |  | 4522 | 0.708 | -0.0068 | No |
| 25 | EGF | na |  |  | 4542 | 0.703 | -0.0017 | No |
| 26 | SLC12A2 | na |  |  | 4547 | 0.702 | 0.0042 | No |
| 27 | INCENP | na |  |  | 4652 | 0.685 | 0.0047 | No |
| 28 | MYBL2 | na |  |  | 4657 | 0.683 | 0.0104 | No |
| 29 | SMC1A | na |  |  | 4737 | 0.670 | 0.0120 | No |
| 30 | MARCKS | na |  |  | 4831 | 0.652 | 0.0128 | No |
| 31 | TRA2B | na |  |  | 4957 | 0.634 | 0.0117 | No |
| 32 | TACC3 | na |  |  | 5096 | 0.614 | 0.0098 | No |
| 33 | ESPL1 | na |  |  | 5108 | 0.612 | 0.0145 | No |
| 34 | KIF22 | na |  |  | 5147 | 0.606 | 0.0178 | No |
| 35 | SLC7A5 | na |  |  | 5187 | 0.602 | 0.0209 | No |
| 36 | CDC25A | na |  |  | 5482 | 0.555 | 0.0103 | No |
| 37 | MCM2 | na |  |  | 5595 | 0.539 | 0.0091 | No |
| 38 | AURKB | na |  |  | 5662 | 0.530 | 0.0102 | No |
| 39 | PML | na |  |  | 5893 | 0.500 | 0.0024 | No |
| 40 | ATF5 | na |  |  | 5960 | 0.491 | 0.0032 | No |
| 41 | CDKN1B | na |  |  | 6043 | 0.481 | 0.0031 | No |
| 42 | TRAIP | na |  |  | 6087 | 0.473 | 0.0049 | No |
| 43 | CCNF | na |  |  | 6105 | 0.470 | 0.0081 | No |
| 44 | CDKN2C | na |  |  | 6116 | 0.469 | 0.0117 | No |
| 45 | MAPK14 | na |  |  | 6223 | 0.455 | 0.0100 | No |
| 46 | EZH2 | na |  |  | 6297 | 0.445 | 0.0100 | No |
| 47 | CDC45 | na |  |  | 6492 | 0.417 | 0.0035 | No |
| 48 | POLQ | na |  |  | 6533 | 0.412 | 0.0049 | No |
| 49 | TROAP | na |  |  | 6635 | 0.403 | 0.0031 | No |
| 50 | PRPF4B | na |  |  | 6877 | 0.374 | -0.0063 | No |
| 51 | DTYMK | na |  |  | 6912 | 0.367 | -0.0049 | No |
| 52 | SLC7A1 | na |  |  | 7059 | 0.353 | -0.0096 | No |
| 53 | CUL4A | na |  |  | 7184 | 0.341 | -0.0131 | No |
| 54 | SRSF10 | na |  |  | 7367 | 0.316 | -0.0199 | No |
| 55 | CCND1 | na |  |  | 7494 | 0.308 | -0.0239 | No |
| 56 | TFDP1 | na |  |  | 7738 | 0.282 | -0.0342 | No |
| 57 | FANCC | na |  |  | 7810 | 0.271 | -0.0356 | No |
| 58 | CDC7 | na |  |  | 8191 | 0.234 | -0.0536 | No |
| 59 | UBE2S | na |  |  | 8271 | 0.223 | -0.0558 | No |
| 60 | BARD1 | na |  |  | 8323 | 0.217 | -0.0566 | No |
| 61 | H2AFX | na |  |  | 8328 | 0.216 | -0.0550 | No |
| 62 | MCM5 | na |  |  | 8383 | 0.209 | -0.0560 | No |
| 63 | NOTCH2 | na |  |  | 8394 | 0.208 | -0.0547 | No |
| 64 | CDC6 | na |  |  | 8605 | 0.191 | -0.0641 | No |
| 65 | ODC1 | na |  |  | 8852 | 0.159 | -0.0757 | No |
| 66 | NOLC1 | na |  |  | 9142 | 0.135 | -0.0897 | No |
| 67 | HIRA | na |  |  | 9177 | 0.129 | -0.0904 | No |
| 68 | LMNB1 | na |  |  | 9190 | 0.127 | -0.0899 | No |
| 69 | PAFAH1B1 | na |  |  | 9194 | 0.126 | -0.0889 | No |
| 70 | GINS2 | na |  |  | 9453 | 0.096 | -0.1017 | No |
| 71 | CDC20 | na |  |  | 9617 | 0.086 | -0.1095 | No |
| 72 | MCM3 | na |  |  | 9637 | 0.083 | -0.1098 | No |
| 73 | YTHDC1 | na |  |  | 9706 | 0.076 | -0.1127 | No |
| 74 | E2F3 | na |  |  | 9893 | 0.056 | -0.1220 | No |
| 75 | TGFB1 | na |  |  | 9937 | 0.048 | -0.1239 | No |
| 76 | LIG3 | na |  |  | 10471 | -0.002 | -0.1519 | No |
| 77 | BCL3 | na |  |  | 10566 | -0.012 | -0.1567 | No |
| 78 | HNRNPU | na |  |  | 10586 | -0.015 | -0.1576 | No |
| 79 | EWSR1 | na |  |  | 10668 | -0.026 | -0.1616 | No |
| 80 | NUP50 | na |  |  | 10727 | -0.032 | -0.1644 | No |
| 81 | CASP8AP2 | na |  |  | 10789 | -0.036 | -0.1673 | No |
| 82 | EFNA5 | na |  |  | 10800 | -0.037 | -0.1675 | No |
| 83 | PLK1 | na |  |  | 10895 | -0.053 | -0.1720 | No |
| 84 | MEIS1 | na |  |  | 10978 | -0.063 | -0.1757 | No |
| 85 | STAG1 | na |  |  | 11103 | -0.080 | -0.1816 | No |
| 86 | SMC4 | na |  |  | 11306 | -0.090 | -0.1914 | No |
| 87 | MAD2L1 | na |  |  | 11353 | -0.097 | -0.1930 | No |
| 88 | KIF4A | na |  |  | 11399 | -0.101 | -0.1945 | No |
| 89 | SRSF1 | na |  |  | 11410 | -0.102 | -0.1941 | No |
| 90 | RBL1 | na |  |  | 11426 | -0.104 | -0.1940 | No |
| 91 | PURA | na |  |  | 11496 | -0.110 | -0.1967 | No |
| 92 | EXO1 | na |  |  | 11505 | -0.112 | -0.1961 | No |
| 93 | CBX1 | na |  |  | 11686 | -0.137 | -0.2044 | No |
| 94 | CDKN3 | na |  |  | 11708 | -0.139 | -0.2043 | No |
| 95 | MNAT1 | na |  |  | 11709 | -0.139 | -0.2031 | No |
| 96 | MCM6 | na |  |  | 11728 | -0.142 | -0.2028 | No |
| 97 | CUL5 | na |  |  | 11886 | -0.161 | -0.2097 | No |
| 98 | DKC1 | na |  |  | 11919 | -0.166 | -0.2100 | No |
| 99 | KIF2C | na |  |  | 11924 | -0.166 | -0.2087 | No |
| 100 | SNRPD1 | na |  |  | 11926 | -0.166 | -0.2073 | No |
| 101 | KIF15 | na |  |  | 12046 | -0.179 | -0.2120 | No |
| 102 | BUB1 | na |  |  | 12059 | -0.180 | -0.2111 | No |
| 103 | HMGN2 | na |  |  | 12062 | -0.181 | -0.2096 | No |
| 104 | KIF23 | na |  |  | 12161 | -0.194 | -0.2131 | No |
| 105 | ARID4A | na |  |  | 12181 | -0.198 | -0.2124 | No |
| 106 | TPX2 | na |  |  | 12233 | -0.204 | -0.2133 | No |
| 107 | ORC6 | na |  |  | 12622 | -0.237 | -0.2317 | No |
| 108 | SUV39H1 | na |  |  | 12643 | -0.239 | -0.2306 | No |
| 109 | SMARCC1 | na |  |  | 12750 | -0.252 | -0.2340 | No |
| 110 | RAD23B | na |  |  | 12787 | -0.256 | -0.2337 | No |
| 111 | NUSAP1 | na |  |  | 12808 | -0.258 | -0.2325 | No |
| 112 | PRIM2 | na |  |  | 12834 | -0.262 | -0.2316 | No |
| 113 | CENPF | na |  |  | 12914 | -0.273 | -0.2333 | No |
| 114 | CUL3 | na |  |  | 12990 | -0.282 | -0.2348 | No |
| 115 | WRN | na |  |  | 13165 | -0.309 | -0.2413 | No |
| 116 | KIF11 | na |  |  | 13273 | -0.320 | -0.2442 | No |
| 117 | MEIS2 | na |  |  | 13451 | -0.345 | -0.2505 | No |
| 118 | KIF5B | na |  |  | 13478 | -0.350 | -0.2488 | No |
| 119 | RACGAP1 | na |  |  | 13570 | -0.365 | -0.2505 | No |
| 120 | NCL | na |  |  | 13574 | -0.366 | -0.2474 | No |
| 121 | CTCF | na |  |  | 13744 | -0.395 | -0.2529 | Yes |
| 122 | BUB3 | na |  |  | 13790 | -0.401 | -0.2518 | Yes |
| 123 | PLK4 | na |  |  | 13826 | -0.410 | -0.2501 | Yes |
| 124 | MTF2 | na |  |  | 13832 | -0.411 | -0.2468 | Yes |
| 125 | CHEK1 | na |  |  | 13855 | -0.412 | -0.2444 | Yes |
| 126 | DR1 | na |  |  | 13996 | -0.415 | -0.2481 | Yes |
| 127 | NASP | na |  |  | 14013 | -0.418 | -0.2453 | Yes |
| 128 | KIF20B | na |  |  | 14040 | -0.422 | -0.2430 | Yes |
| 129 | NUP98 | na |  |  | 14128 | -0.430 | -0.2439 | Yes |
| 130 | STIL | na |  |  | 14238 | -0.449 | -0.2457 | Yes |
| 131 | DBF4 | na |  |  | 14298 | -0.463 | -0.2448 | Yes |
| 132 | LBR | na |  |  | 14304 | -0.464 | -0.2410 | Yes |
| 133 | BRCA2 | na |  |  | 14335 | -0.469 | -0.2386 | Yes |
| 134 | AURKA | na |  |  | 14577 | -0.502 | -0.2469 | Yes |
| 135 | BIRC5 | na |  |  | 14629 | -0.512 | -0.2451 | Yes |
| 136 | G3BP1 | na |  |  | 14666 | -0.518 | -0.2425 | Yes |
| 137 | KPNB1 | na |  |  | 14673 | -0.519 | -0.2383 | Yes |
| 138 | FOXN3 | na |  |  | 14685 | -0.520 | -0.2344 | Yes |
| 139 | CUL1 | na |  |  | 14693 | -0.522 | -0.2302 | Yes |
| 140 | CCNA2 | na |  |  | 14794 | -0.537 | -0.2309 | Yes |
| 141 | TMPO | na |  |  | 14804 | -0.538 | -0.2267 | Yes |
| 142 | PBK | na |  |  | 15143 | -0.588 | -0.2393 | Yes |
| 143 | DMD | na |  |  | 15243 | -0.605 | -0.2393 | Yes |
| 144 | SAP30 | na |  |  | 15285 | -0.614 | -0.2361 | Yes |
| 145 | ATRX | na |  |  | 15309 | -0.618 | -0.2320 | Yes |
| 146 | GSPT1 | na |  |  | 15339 | -0.624 | -0.2281 | Yes |
| 147 | STMN1 | na |  |  | 15496 | -0.649 | -0.2307 | Yes |
| 148 | H2AFZ | na |  |  | 15730 | -0.688 | -0.2370 | Yes |
| 149 | CENPE | na |  |  | 15857 | -0.715 | -0.2374 | Yes |
| 150 | NDC80 | na |  |  | 15904 | -0.723 | -0.2335 | Yes |
| 151 | UBE2C | na |  |  | 15936 | -0.731 | -0.2288 | Yes |
| 152 | RPS6KA5 | na |  |  | 15965 | -0.737 | -0.2239 | Yes |
| 153 | TOP2A | na |  |  | 15987 | -0.741 | -0.2186 | Yes |
| 154 | AMD1 | na |  |  | 16032 | -0.755 | -0.2144 | Yes |
| 155 | PRC1 | na |  |  | 16085 | -0.766 | -0.2104 | Yes |
| 156 | RAD21 | na |  |  | 16290 | -0.809 | -0.2142 | Yes |
| 157 | SMC2 | na |  |  | 16293 | -0.809 | -0.2072 | Yes |
| 158 | SQLE | na |  |  | 16311 | -0.813 | -0.2011 | Yes |
| 159 | HMMR | na |  |  | 16468 | -0.845 | -0.2020 | Yes |
| 160 | H2AFV | na |  |  | 16477 | -0.848 | -0.1950 | Yes |
| 161 | SRSF2 | na |  |  | 16515 | -0.858 | -0.1895 | Yes |
| 162 | PDS5B | na |  |  | 16521 | -0.859 | -0.1824 | Yes |
| 163 | CCNT1 | na |  |  | 16766 | -0.917 | -0.1872 | Yes |
| 164 | CDK1 | na |  |  | 16949 | -0.970 | -0.1884 | Yes |
| 165 | CDK4 | na |  |  | 16963 | -0.974 | -0.1806 | Yes |
| 166 | XPO1 | na |  |  | 16967 | -0.975 | -0.1723 | Yes |
| 167 | CENPA | na |  |  | 17024 | -0.993 | -0.1667 | Yes |
| 168 | CCNB2 | na |  |  | 17048 | -0.999 | -0.1592 | Yes |
| 169 | CKS1B | na |  |  | 17139 | -1.027 | -0.1550 | Yes |
| 170 | MT2A | na |  |  | 17171 | -1.038 | -0.1477 | Yes |
| 171 | SLC38A1 | na |  |  | 17286 | -1.078 | -0.1443 | Yes |
| 172 | KPNA2 | na |  |  | 17340 | -1.098 | -0.1376 | Yes |
| 173 | TOP1 | na |  |  | 17369 | -1.108 | -0.1294 | Yes |
| 174 | PTTG1 | na |  |  | 17418 | -1.121 | -0.1222 | Yes |
| 175 | HMGB3 | na |  |  | 17427 | -1.125 | -0.1129 | Yes |
| 176 | HIST1H2BK | na |  |  | 17553 | -1.178 | -0.1093 | Yes |
| 177 | ORC5 | na |  |  | 17666 | -1.228 | -0.1045 | Yes |
| 178 | SYNCRIP | na |  |  | 17710 | -1.247 | -0.0959 | Yes |
| 179 | FBXO5 | na |  |  | 17725 | -1.253 | -0.0858 | Yes |
| 180 | NEK2 | na |  |  | 17957 | -1.352 | -0.0862 | Yes |
| 181 | PRMT5 | na |  |  | 18005 | -1.386 | -0.0767 | Yes |
| 182 | HNRNPD | na |  |  | 18008 | -1.388 | -0.0648 | Yes |
| 183 | RPA2 | na |  |  | 18162 | -1.475 | -0.0600 | Yes |
| 184 | HIF1A | na |  |  | 18348 | -1.603 | -0.0558 | Yes |
| 185 | HUS1 | na |  |  | 18375 | -1.622 | -0.0431 | Yes |
| 186 | CDC27 | na |  |  | 18397 | -1.639 | -0.0300 | Yes |
| 187 | CKS2 | na |  |  | 18444 | -1.669 | -0.0180 | Yes |
| 188 | SS18 | na |  |  | 18730 | -1.926 | -0.0163 | Yes |
| 189 | TTK | na |  |  | 18742 | -1.945 | 0.0000 | Yes |
| 190 | KATNA1 | na |  |  | 19102 | -2.756 | 0.0051 | Yes |
Table: GSEA details [plain text format]

  

Fig 2: HALLMARK\_G2M\_CHECKPOINT      
 Blue-Pink O' Gram in the Space of the Analyzed GeneSet

  

Fig 3: HALLMARK\_G2M\_CHECKPOINT: Random ES distribution      
 Gene set null distribution of ES for **HALLMARK\_G2M\_CHECKPOINT**
